# Studies on Prn Variation in the Mouse Model and Comparison with Epidemiological Data

**DOI:** 10.1371/journal.pone.0018014

**Published:** 2011-03-25

**Authors:** Marjolein van Gent, Inge H. M. van Loo, Kees J. Heuvelman, Albert J. de Neeling, Peter Teunis, Frits R. Mooi

**Affiliations:** 1 Laboratory for Infectious Diseases and Screening, Centre for Infectious Disease Control, National Institute for Public Health and the Environment, Bilthoven, The Netherlands; 2 Department of Medical Microbiology, Maastricht University Medical Centre, Maastricht, The Netherlands; 3 Expertise Centre for Methodology and Information Services, National Institute for Public Health and the Environment, Bilthoven, The Netherlands; Fundació Institut Germans Trias i Pujol; Universitat Autònoma de Barcelona CibeRES, Spain

## Abstract

The virulence factor pertactin (Prn) is a component of pertussis vaccines and one of the most polymorphic *Bordetella pertussis* antigens. After the introduction of vaccination shifts in predominant Prn types were observed and strains with the Prn vaccine type (Prn1) were replaced by strains carrying non-vaccine types (Prn2 and Prn3), suggesting vaccine-driven selection. The aim of this study was to elucidate the shifts observed in Prn variants. We show that, although Prn2 and Prn3 circulated in similar frequencies in the 1970s and 1980s, in the 1990s Prn2 strains expanded and Prn3 strains disappeared, suggesting that in vaccinated populations Prn2 strains are fitter than Prn3 strains. We established a role for Prn in the mouse model by showing that a Prn knock-out (Prn-ko) mutation reduced colonization in trachea and lungs. Restoration of the mutation resulted in a significant increase in colonization compared to the knock-out mutant. The ability of clinical isolates with different Prn variants to colonize the mouse lung was compared. Although these isolates were also polymorphic at other loci, only variation in the promoter for pertussis toxin (*ptxP*) and Prn were found to contribute significantly to differences in colonization. Analysis of a subset of strains with the same *ptxP* allele revealed that the ability to colonize mice decreased in the order Prn1>Prn2 and Prn3. Our results are consistent with the predominance of Prn1 strains in unvaccinated populations. Our results show that ability to colonize mice is practically the same for Prn2 and Prn3. Therefore other factors may have contributed to the predominance of Prn2 in vaccinated populations. The mouse model may be useful to assess and predict changes in the *B. pertussis* population due to vaccination.

## Introduction

*Bordetella pertussis* causes whooping cough or pertussis, a respiratory disease that is most severe in infants. Before childhood vaccination was introduced in the 1950s, pertussis was a major cause of infant mortality worldwide. Whole cell vaccines against pertussis were introduced in the 1940s to 1960s and these were replaced by more defined and less reactogenic acellular vaccines (ACVs) in the 1990s [1], [2]. All ACVs contain pertussis toxin (Ptx). In addition, they may contain filamentous hemagglutinin (FHA), pertactin (Prn) and serotype 2 and 3 fimbriae. Widespread vaccination of children significantly reduced morbidity and mortality. However, in the 1990s a resurgence of pertussis was observed in countries with highly vaccinated populations and pertussis has become the most prevalent vaccine preventable disease in developed countries [2], [3]. Although morbidity is highest in newly born, pertussis is now recognized as a frequent infection of adults [4], [5].

The re-emergence of pertussis has been attributed to waning vaccine-induced immunity and pathogen adaptation [3], [6]. Pathogen adaptation is supported by several observations. Antigenic divergence has occurred between vaccine strains and clinical isolates with respect to several vaccine components; Ptx, Prn, and fimbriae [3], [6], [7], [8]. Further variation in Ptx and Prn has been shown to affect vaccine efficacy in a mouse model [9], [10], [11], [12], [13]. In addition to antigenic variation, increased Ptx production has been associated with the resurgence of pertussis [14]. Strains with a novel allele for the Ptx promoter (*ptxP3*) emerged in the 1990s, replacing the resident *ptxP1* strains. A role of vaccination in driving shifts in *B. pertussis* populations is also supported by recent genomic data [15], [16].

**Figure 1:**
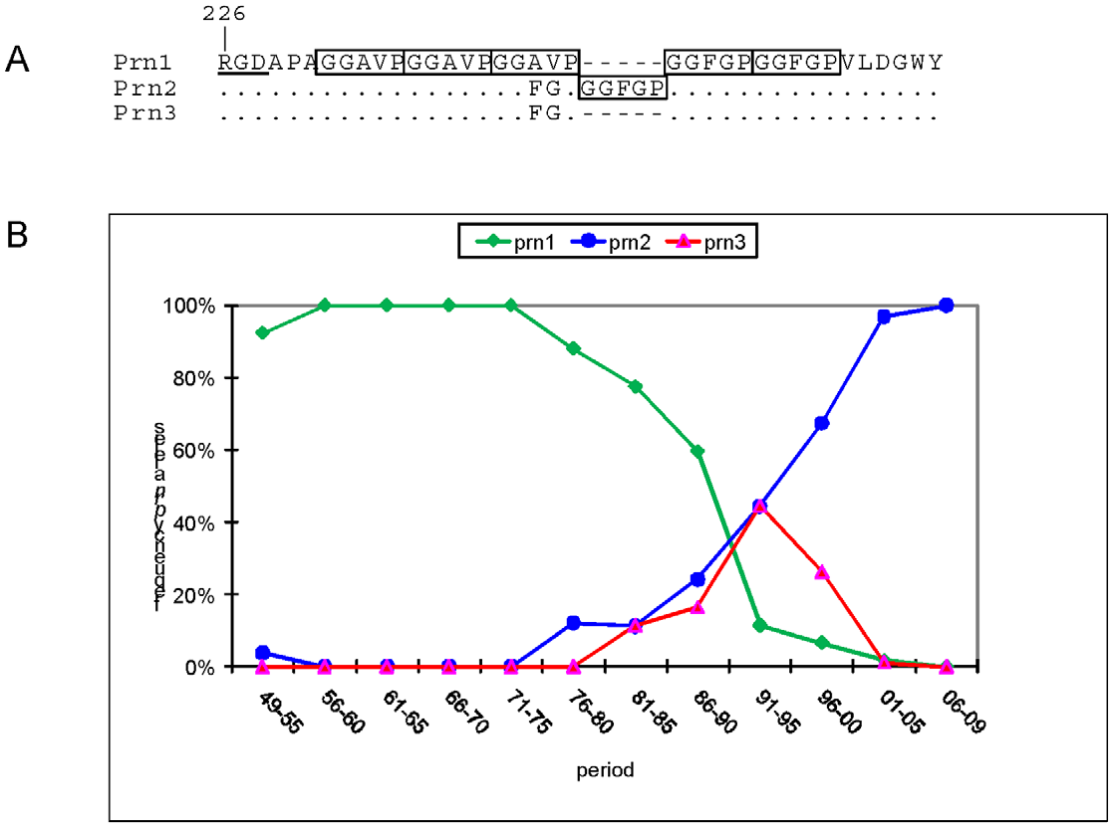
Variation in pertactin (A) and temporal trends in the frequency of pertactin alleles (B). The number in the pertactin sequences indicates the amino acid position relative to the N-terminus. The RGD motif implicated in binding to host receptors has been underlined. Repeat motifs are blocked. Dots and dashes indicate sequence identity with Prn1 and gaps, respectively. No strains were available from the period 1960-1964 to determine allele frequencies. Vaccination against pertussis was introduced in 1953.

Prn, the focus of this study, is one of the most polymorphic *B. pertussis* proteins known and 13 *prn* alleles have been identified so far [3], [6]. Variation in Prn is mainly limited to two regions, designated R1 and R2, which are comprised of Gly-Gly-X-X-Pro and Pro-Gln-Pro repeats, respectively. The R1 region is located proximal to a RGD motif implicated in receptor binding [17] (Fig. 1A). Studies in a number of countries have revealed similar temporal trends in the frequency of Prn variants [8], [10], [18], [19], [20], [21], [22], [23], [24], [25], [26], [27], [28]. In the last fifty years three Prn variants have been found to predominate: Prn1, Prn2 and to a lesser extent Prn3. In the prevaccination era, essentially all analyzed strains produced Prn1. However in the 1980s, 20 to 30 years after the introduction of whole cell vaccination, Prn strains were replaced by Prn2 and Prn3 strains. As most vaccines contain Prn1 it was suggested that the emergence of Prn2 and Prn3 was vaccine-driven [8]. Consistent with this assumption, it was found that a whole cell vaccine containing Prn1 was less effective against Prn2 strains compared to Prn1 strains in a mouse model [13]. Prn2 strains now predominate in most vaccinated populations. Njamkepo et al. [29] have analyzed strains from a region in Senegal where vaccination was introduced only recently (and where the coverage was still low). They found that, in contrast to strains isolated in the same period in France (where vaccination was introduced in the 1950s and where vaccination coverage is high), all Senegalese strains carried the *prn1* allele, characteristic for prevaccination strains. This observation is consistent with the theory of vaccine-driven shifts. Other arguments for the role of vaccination in shifts of Prn variants have recently been reviewed [3]. In the 1990s, whole cell vaccines were replaced by acellular vaccines. Sporadically, in the acellular vaccine period, strains have been isolated which do not produce Prn [30]. However, recently in France a significant percentage (5.6%) of the strains isolated from hospitalized children did not produce pertactin [31]. It was found that the Prn gene was inactivated by deletion or insertion of IS481 [31]. Spread of such Prn-knock out strains may reduce vaccine efficacy, in particular of acellular vaccines which induce a less broad immunity than whole cell vaccines. Evaluation of this threat is hampered by our lack of understanding of the role Prn plays in the ecology of *B. pertussis*. Here we compare the ability of a Prn-knock out strain with a wild type strain to colonize the mouse lungs and trachea. We also assess the effect of variation in R1 of Prn on colonization in this model.

## Methods

### Bacterial strains

*B. pertussis* strains were isolated from Dutch patients in the period 1949 to 1996. *S*trains were grown on Bordet-Gengou agar (Difco Catalogue no. 0048-17-5) supplemented with 1% (v/v) glycerol and 15% (v/v) sheep blood at 35 °C for 3 days. Strains were made streptomycin resistant to allow recovery on selective plates from mouse lungs and trachea. Characteristics of the strains used are shown in Table S1. Tohama I derivatives were used to construct Prn mutants [32] (Table 1).

**Table 1:** Characteristics of strains used in this study^1^.

Tohama I derivatives			
Strain designation:	Genotype:	Remarks:	Reference:
B0213	*ptxP1, ptxA2, tcfA2, prn1,* Fim2	Strep resistant Tohama I derivative	[48]
B1686	*ptxP1, ptxA2, tcfA2, prn::kan,* Fim2	Prn knock out derived from B0213	This work
B2576	*ptxP1, ptxA2, tcfA2, prn1,* Fim2	Back mutant derived from B1686	This work

Vaccine strains			
Strain designation:	Genotype:	Remarks:	Reference:
Strain 134	*ptxP1, ptxA2, tcfA2, prn1,* Fim2,3	Used for the Dutch whole-cell vaccine	[3]
Strain 509	*ptxP1, ptxA4, tcfA2, prn7,* Fim2,3	Used for the Dutch whole-cell vaccine	[3]

1 Alleles for *ptxP, ptxA, tcfA* and *prn* are shown in addition to the fimbrial (Fim) serotype produced. A table with clinical isolates is provided (Table S1).

### Construction of Prn knock-out mutants

To construct a Prn knockout mutant (Prn*-*ko), a fragment of *prn* (469 bases in size) was amplified using primers Prn*Xba*1F (GCTCTAGAGCCTGGCATCCAATGAACATGT) and Prn*EcoR*1R (GGAATTCCTGTTCGCCGGCCACATAG), and cloned into pSS1129 [33], resulting in pSR2.1. Subsequently, a kanamycin resistance gene cassette was cloned into the RsrII site located in the 469 base *prn* fragment of pSR2.1, resulting in pSR2-1.1. The latter plasmid was used to introduce the *kan* gene into *prn1* of the Tohama I derivative B213 by allelic exchange [34]. Correct insertion was checked by sequencing and one strain, B1686, was selected for further studies. Expression of Prn in the knock-out mutant was restored by allelic exchange using pSR2.1. Back mutants were identified by PCR and DNA sequencing. One clone, B2576, was selected for studies in the mouse model. Expression of Prn by B213 and its derivatives was assessed by immunoblotting.

### Strain typing

Genotyping was focused on the following genes essentially as described in [35], [36]: *prn*, *ptxP*, *ptxA* and *tcfA*, coding for, respectively, Prn, the Ptx promoter, the Ptx S1 subunit, and the tracheal colonization factor. Typing of *prn* alleles is done by sequencing of the repeat regions 1 and 2, and does not distinguish between *prn1* and *prn7* which are identical in region 1 and 2 but differ in a non-silent SNP outside these regions. Whole genome sequencing has revealed that *prn7* is associated with strains which only circulated in the 1950s and 1960s and we assumed here that all strains harbored *prn1.* Serotyping, which distinguishes between strains producing type 2 or type 3 fimbriae (Fim2 or Fim3) was carried out using the slide agglutination technique [37].

### Mouse infection model

All animal was conducted according to relevant national and international guidelines. Strains were grown on Bordet-Gengou plates at 35°C for 24 hrs. After harvesting, the bacterial concentration was adjusted to 5×10^9^ bacteria/ml. The bacterial suspensions were flash-frozen in ethanol/dry ice in small aliquots in Verwey medium [38] with 15% glycerol and stored at –80°C. The viability of the frozen cell suspensions was determined prior to infection. For infection, eight BALB/c mice (Harlan, OlHsd) 4–5 weeks old, were lightly anaesthetised with isoflurane and 40 µl of the inoculum, containing 2*10^7^ CFUs bacteria, was placed on the nostrils and allowed to be inhaled. Three days after infection, mice were sacrificed by intraperitoneal injection of Nembutal^R^ (Sanofi/Algrin). To recover *B. pertussis,* trachea and lungs were removed. The trachea was vortexed in 500 µl Verwey medium with 5 glass pearls for 30 sec at RT. Lungs were homogenised in 900 µl Verwey medium for 10 sec at 20,000 rpm with a homogenizer (Pro Scientific, Pro200) at RT. Appropriate dilutions were plated on Bordet-Gengou plates supplemented with streptomycin, and the number of CFUs was determined. All animal experiments were approved by the Institute's Animal Ethics Committee.

### Statistical analysis

CFU counts recovered from mice were log-transformed and zero counts were considered missing values. The data were analysed by Proc GLM in SAS 9.1 (SAS Institute Inc., Cary, NC, USA). The significance among means was tested by the Student-Newman-Keuls test at alpha  = 0.01 or 0.05. Strengths of effects of variation in fimbrial serotype, Prn, the Ptx promoter (*ptxP*), the Ptx S1 subunit (PtxA) and TcfA on colonization were assessed by ANOVA, followed by a Tukey post-test (Tukey Honest Significant Differences). The ANOVA table can be found in the supporting information section (Table S2). Based on this information, the effect of variation in Prn was assessed in a subset of strains which harbored the same *ptxP* allele, *ptxP1*.

## Results

### Temporal trends in *prn* alleles in the Netherlands

In a previous work, we analyzed the frequencies of Prn variants in the Netherlands between 1949 and 1996 [8]. Here we extended this analysis to 2009 and included more strains. Pertussis vaccination in the Netherlands was introduced in 1953. The strain composition of the whole cell vaccine was changed a few times, but remained the same from the early 1960s on, when it was comprised of two strains producing Prn1 and Prn7. Prn1 and Prn7 are identical, except for a single amino acid substitution outside the two repeat regions [3]. In the period 1949–1975, Prn1 strains predominated, representing 97% of the isolates (N 64) (Fig. 1B). In this period only two other Prn variants were observed, each in a single strain, Prn2 in 1950 and Prn10 in 1954. In the period 1976–1985, Prn2 and Prn3 strains emerged. Initially, frequencies of both Prn2 and Prn3 strains increased with similar rates. However, in the mid 1990s Prn3 strains decreased in frequency, while Prn2 strains continued to expand. Since 2003, only Prn2 strains have been detected in the Netherlands (N 203). These data suggest that Prn1 strains are more fit in unvaccinated populations, while the non-vaccine types, Prn2 and Prn3, strain are more fit in vaccinated populations. However, of the two non-vaccine types, Prn2 seemed to confer the greatest increase in fitness in vaccinated populations.

### The effect of inactivation of the *prn* gene on colonization of the mouse respiratory tract

**Figure 2:**
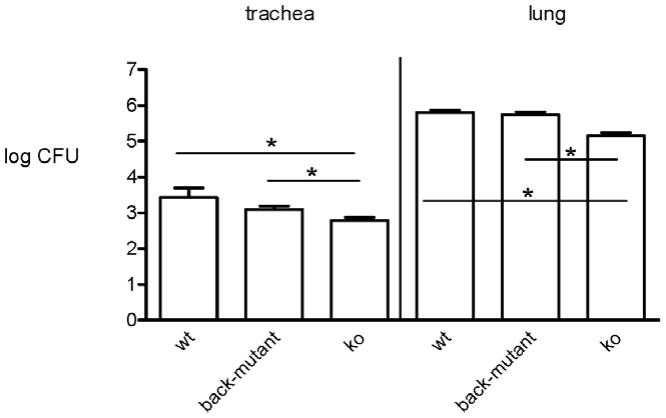
Role of Prn in colonization of the mouse respiratory tract. Mice were infected intranasally with the Tohama I strain (wt), a mutant derivative in which the Prn was inactivated (ko), or a back-mutant in which the Prn gene was restored. Three days post-infection, CFUs were determined in trachea and lungs. The experiment was performed twice, and pooled data from both experiments are shown. Error bars and asterisks indicate 95% confidence intervals and significant differences (P<0.01), respectively.

In a next step we aimed to determine the effect of variation in Prn on colonization of the mouse respiratory tract. However, it was not clear whether Prn played any role in this animal model. To investigate this, mice were infected with the Tohama I strain or a derivative in which the Prn gene was inactivated by insertion of a kanamycin resistant gene cassette (Prn-ko strain). Subsequently, colonization was assessed three days after infection (Fig. 2). The Prn-ko mutation reduced colonization in trachea and lungs 6- and 4-fold, respectively (P<0.01). When the wild type phenotype was restored by back mutating the knock-out strain, colonization of trachea and lungs was restored (Fig. 2). Although the back mutant showed slightly lower colonization levels compared to the wild type strain in trachea and lungs, the difference was not statistically significant. In contrast, the back mutation significantly increased colonization compared to the knock-out mutant in trachea and lungs (P<0.01).

### The effect of natural variation in Prn on colonization of the mouse lung

**Figure 3:**
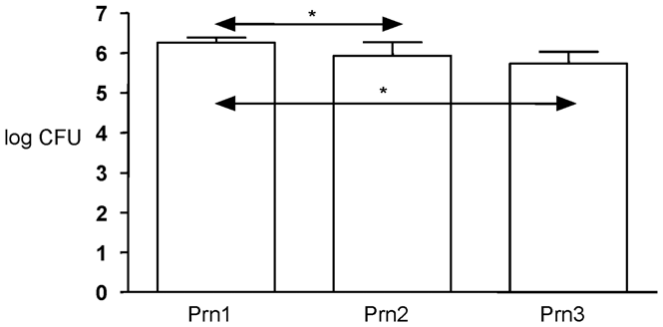
Effect of variation in Prn on colonization of the mouse lung. Mice were infected with clinical isolates producing Prn1 (N = 15), Prn2 (N = 5) or Prn3 (N = 8). Three days after infection, the number of CFUs in the lung was determined. Error bars and asterisks indicate 95% confidence intervals and significant differences (P<0.05), respectively.

To investigate the effect of natural variation in Prn on colonization, mice were infected with clinical isolates producing one of the three predominant Prn variants, Prn1, Prn2 or Prn3. Colonization of lungs was assessed three days post infection. A comparison of the colonizing ability of Prn variants was complicated by the fact that, in addition to Prn, the strains showed variation in a number of other surface proteins such as PtxA, TcfA and fimbriae (Table S1). Further, strains also differed with respect to the pertussis toxin promoter, *ptxP*. Three *ptxP* alleles (*ptxP1*, *ptxP2* and *ptxP3*) were present in this collection of strains. We have shown that *ptxP3* strains produce more Ptx than *ptxP1* [14] and Ptx has been shown to affect the colonization of naïve mice [39]. Thus any effect observed in mice could be due to one or more of these polymorphic loci. Multivariate analyses revealed that only variation in Prn and *ptxP* significantly affected colonization of the mouse (P 0.007 and 0.024, respectively). Based on this information, the effect of variation in Prn was assessed in a subset of strains which harbored the same *ptxP* allele, *ptxP1*. Only *ptxP1* strains were used in these experiments because the changes observed in Prn types occurred in a period when most strains carried the *ptxP1* allele. It was found that the ability of the strains to colonize the mouse lung decreased in the order Prn1>Prn2>Prn3 (Fig. 3). Only the difference between Prn1 and Prn2 was significant, however.

## Discussion

The role Prn plays in the ecology of *B. pertussis* is still under investigation. Prn is non-covalently attached to the outer membrane and may play a role in adherence to monocytes and other host cells [17], . Further, Prn may affect the host immune response as it has been shown that it can augment the suppressive effect of FHA on LPS-induced IL-12 production in vitro [41]. The role of Prn in colonization of the mouse respiratory tract has been studied with *B. pertussis* and the closely related species *Bordetella bronchiseptica*. Nicholson and coworkers [42], used a swine isolate of *B. bronchiseptica* to study the contribution of FHA and Prn to respiratory disease in swine. They found that colonization of the FHA-knock out (FHA-ko) mutant was lower than that of the wild type in the nasal cavity, trachea and lungs. Further, the FHA-ko mutant caused limited to no disease. In contrast, the Prn-ko mutant caused similar disease severity relative to the wild type, however, colonization of the Prn mutant was reduced relative to the wild type during early and late infection. Inatsuka and coworkers, studied the role of Prn in both rats and mice [43]. They observed that, while a Prn-ko *B. bronchiseptica* strain did not differ from a wild type strain in its ability to establish respiratory infection in rats, it was cleared much faster than wild type bacteria from the mouse lung. These authors went on to show that Prn allows *B. bronchiseptica* to resist neutrophil-mediated clearance. Studies with *B. pertussis* gave less clear results with respect to the role of Prn. Roberts and coworkers [44] concluded that a Prn mutant was able to colonize and grow in the lungs and trachea of mice as well as the parent strain, BBC26, although it reached slightly lower levels in both organs. In contrast, in two other studies no effect of a Prn mutation on colonization of the mouse lung was found [45], [46]. Overall, these results suggested that the role of Prn in colonization of the mouse was subtle.

In this study, inactivation of the Prn gene significantly reduced colonization of both the trachea and lungs of mice (Fig. 2). Back mutation restored the colonization ability. When clinical isolates were tested for their ability to colonize the mouse lung, significant differences were found. The clinical isolates differ at several other loci making it difficult to assign phenotypes to particular polymorphisms. Multivariate analyses indicated that only variation in *ptxP* and Prn contributed significantly to the differences in colonization. Ptx enhances colonization of the mouse respiratory tract, presumably by suppressing host innate immunity [39]. Thus the effect of *ptxP* on colonization is probably due to different levels of Ptx produced in vivo, consistent with in vitro data [14]. When strains were compared which carried an identical *ptxP* allele (*ptxP1*), it was observed that the ability to colonize mice decreased in the order Prn1>Prn2 and Prn3 (Fig. 3). This may be due to differential binding of Prn variants to host receptors, as the variable region is located close to the RGD sequence implicated in attachment [17], [47] (Fig. 1A).

The effect of variation in Prn on colonization of mice lungs shows interesting parallels with epidemiological data. In the prevaccination era, essentially all strains analyzed produced Prn1. Prn2 and Prn3 emerged in the 1980s, 20 to 30 years after the introduction of vaccination and although the frequency of the two variants was initially similar, Prn2 is now by far the most predominant type. Coexistence of Prn1, Prn2 and Prn3 strains in vaccinated populations has also been observed in Finland, Sweden and the UK [28], [48], [49], [50]. Invariably, Prn2 strains rose to predominance. These data suggest that Prn1 strains are more fit in unvaccinated populations, while the non-vaccine types, Prn2 and Prn3, strain are more fit in vaccinated populations. However, of the two non-vaccine types, Prn2 seemed to confer the greatest increase in fitness in vaccinated populations.

Taking together, based on our results and those on vaccinated mice previously published [9], [10], [11], [12], [13] we propose that of the three Prn variants, Prn1 binds most efficiently to the host cells, explaining its predominance in unvaccinated populations. Vaccination with Prn1-containing vaccines may have shifted the competitive balance between Prn variants allowing non-vaccine types Prn2 and Prn3 to emerge. Regarding Prn2 strains, it is not so clear that they colonized more efficiently than Prn3 strains; however, other factors may have contributed to the predominance of Prn2 strains in vaccinated populations. E.g. it is conceivable that antibodies induced by Prn1 bind less well to Prn2 compared to Prn3. Indeed the Dutch WCV was shown to be less efficacious in the mouse model against Prn2 strains compared to Prn3 strains, although the difference was not statistically significant [13]. In addition to Prn1, Prn2 and Prn3, 10 other Prn types have been found in low frequencies in a number of countries [3]. We speculate that the frequency of these variants is the compound effect of receptor fit and cross immunity with vaccine-induced Prn antibodies.

The effect of the Prn-KO mutation in the mouse model was relatively small, and one should be careful in extrapolating these results to human populations. Continued strain surveillance in human populations is required to address the question whether Prn-ko mutants which have emerged in France [31] are less fit in human populations and will have a limited ability to spread or cause disease compared to wild type strains. The *B. pertussis* genome contains many silent genes with unknown function [32] and it is conceivable that compensatory mutations may occur in Prn-ko mutants by gene reactivation or by other mechanisms.

In addition to variation in Prn, variation in *ptxP* was found to significantly affect colonization of the mouse respiratory tract. The long term effect of vaccination on pathogen populations generally cannot be evaluated in the relatively short period in which clinical trials take place. Our results suggest that the mouse model can be used to explain and predict changes in the *B. pertussis* population due to vaccination.

## Supporting Information

Table S1 **Characteristics of the strains used in this study.**
(XLS)

Table S2 **Analyses of variance of the effect of** ***B. pertussis*** **polymorphisms on colonization of the mouse lung.**
(XLS)
